# Tolerance to and Postoperative Outcomes With Early Oral Feeding Following Elective Bowel Surgery: A Systematic Review

**DOI:** 10.7759/cureus.42943

**Published:** 2023-08-04

**Authors:** Lord Mvoula, Evelyn Irizarry

**Affiliations:** 1 Surgery, Lincoln Medical and Mental Health Center, Bronx, USA; 2 Colorectal Surgery, Lincoln Medical and Mental Health Center, Bronx, USA

**Keywords:** intestine, midline incision, open surgery, diet, meals, npo, feeding

## Abstract

Although practice guidelines recommend resuming oral feeding immediately after gastrointestinal surgery, many practitioners remain reluctant to order early oral feeding (EOF). Therefore, this review aimed to clarify the tolerance to and postoperative outcomes with EOF among patients undergoing bowel surgery.

A systematic review of the literature published between January 1990 and July 2022 with the time of oral intake (early or delayed until resolution of ileus) as the exposure variable was conducted using PubMed and Scopus databases. Outcomes of interest included tolerance to EOF and postoperative adverse effects or complications.

After screening 1,667 research articles, 18 randomized control trials, six prospective case series, and four cohort studies met our inclusion criteria, collectively representing data from 2,647 patients in eleven countries. These studies indicate that while most patients tolerate EOF, 5-25% may not tolerate EOF until the fourth postoperative day (POD). Moreover, EOF, at best, has no advantage over delayed feeding in terms of vomiting, nausea, nasogastric tube requirement, or other postoperative complications. In addition, early return of bowel function, lower risk of diarrhea, and lower pain score with EOF are inconsistently reported, and shorter hospitalization with EOF may be limited to those who tolerate oral feeding on POD 0 or 1. Nevertheless, shorter hospitalization with EOF could reduce the cost of hospitalization.

A substantial number of patients may not be able to tolerate oral feeding after bowel surgery until POD 4, and in patients who tolerate EOF, the only clear benefit is a shorter length of hospitalization.

## Introduction and background

Traditional postoperative care for gastrointestinal (GI) surgeries typically involved withholding oral intake until the resolution of ileus indicated by the passage of flatus or stool. Breaking away from the traditional oral feeding (TOF) practice, the American Society for Enhanced Recovery, Perioperative Quality Initiative (POQI), and the European Society for Clinical Nutrition and Metabolism (ESPEN) recommend resumption of oral feeding - including clear liquids, oral nutritional support, and balanced diet-soon after GI surgery to facilitate post-surgery recovery, shorten the length of hospitalization and reduce postoperative morbidity and mortality [1,2]. Resuming oral nutrition immediately after GI surgery is also included as a care element in the Enhanced Recovery After Surgery (ERAS) protocol [3].

These practice guidelines also align with patients’ preferences [4]. For example, when patients who underwent colon resection were advised to resume a regular diet based on their appetite, 70-80% initiated solid meals by the second postoperative day (POD) [5,6]. However, despite the growing scientific consensus and patient preference for early oral feeding (EOF) after GI surgery, many healthcare providers remain reluctant to advise oral intake of fluids or solid meals until the return of bowel function, especially anesthesiologists and general surgeons compared to colorectal surgeons [7-11]. The reluctance is not entirely unfounded: Even though most patients prefer solid meals on POD 1 [12] or 2 [6], only a third may be able to tolerate solid meals by POD 2 [5], and half of the patients by POD 4 [11].

The disconnect between practice guidelines and the healthcare provider’s reluctance to order EOF after elective GI surgeries warrants a review of evidence to ascertain if patients can tolerate EOF and if it produced improved postoperative outcomes compared to TOF for several reasons. Previous systematic reviews have evaluated postoperative outcomes with EOF in patients undergoing any GI surgery [13,14], only lower GI surgery [15,16], or, more specifically, among patients undergoing colorectal surgery [17,18]. Reviews that evaluated the post-surgery outcomes with early feeding to specific surgery sites, such as colon and rectum, were limited by the number of papers meeting the inclusion criteria (n=9 with 879 patients [16], n=7 with 587 patients [17], and n=5 with 985 patients [18]). However, the broad inclusion criteria of some of these reviews led to reporting of the combined outcomes with EOF from several feeding routes - oral, nasojejunal tube, nasoduodenal tube, and jejunostomy [13-15] - or with various surgical sites such as upper GI, lower GI, and hepatobiliary surgery [13,14]. Also, prior reviews do not make a distinction by surgical approach (laparotomy or laparoscopy) even though the resolution of ileus [19] and tolerance to oral feeding [5] are achieved sooner in patients undergoing laparoscopic GI surgery and are more likely to initiate EOF [8,10] compared to a laparotomy procedure.

Therefore, this study aimed to systematically review the tolerance to and postoperative outcomes with EOF among patients undergoing bowel surgery, representing 58% of all GI surgeries worldwide [20]. Moreover, we aimed to separately evaluate the available studies with small or large bowel procedures by surgical approach (laparotomy or laparoscopy).

## Review

Method

This systematic review followed the Preferred Reporting Items for Systematic Reviews and Meta-Analyses (PRISMA) statement [21].

Search Strategy

Our search strategy covered studies indexed in PubMed and Scopus databases between 01 January 1990 and 31 July 2022 to capture the most recent trends (last searched: 05 November 2022) using the following search term: (postoperativ*) AND (rectum OR anus OR colon OR intestine OR bowel) AND (surger* OR colestom* OR resection* OR abdominoperineal) AND (clear fluid* OR oral feed* OR solid feed* OR eat OR eating). The reference lists of earlier systematic reviews and meta-analyses on postoperative feeding practices were also screened.

Study Selection Criteria

Randomized control trials (RCT), prospective case series (PCS), and cohort studies published in English with online access to the full-text article meeting the following criteria were considered for inclusion:

a. Participants ≥18 years had undergone elective bowel surgery primarily to treat an underlying bowel condition.

b. Time to first oral feed (liquid, semi-solid, or solid) or EOF (as defined by the study authors) after surgery was used as exposure variables.

c. Reported tolerance to EOF, adverse effects (e.g., nausea and vomiting), or postoperative complication (including mortality).

Studies with patients who had undergone emergency bowel surgery, palliative bowel surgery, or required prolonged postoperative enteral nutrition through, for instance, a feeding jejunostomy were excluded. Finally, studies reporting the effect of EOF as a part of multimodal recovery programs such as ERAS and “fast-track surgeries” were excluded unless the experimental and control groups varied only by feeding timeline and all other modalities were equally applied to both groups.

Case series and cohort studies were differentiated using the distinction criteria proposed by Mathes and Pieper [22].

Data Extraction and Analysis

Data from eligible studies were tabulated using a predefined form which included study characteristics (author, year of publication, country), population characteristics (sample size, age with standard deviation or range, and gender distribution), surgery characteristics (indication, location, and approach), and the outcomes of interests (tolerance to EOF, postoperative adverse effects, and complications). If the country of the study was not explicitly mentioned, the country of the primary author’s affiliated institution was used.

Results

**Figure 1:**
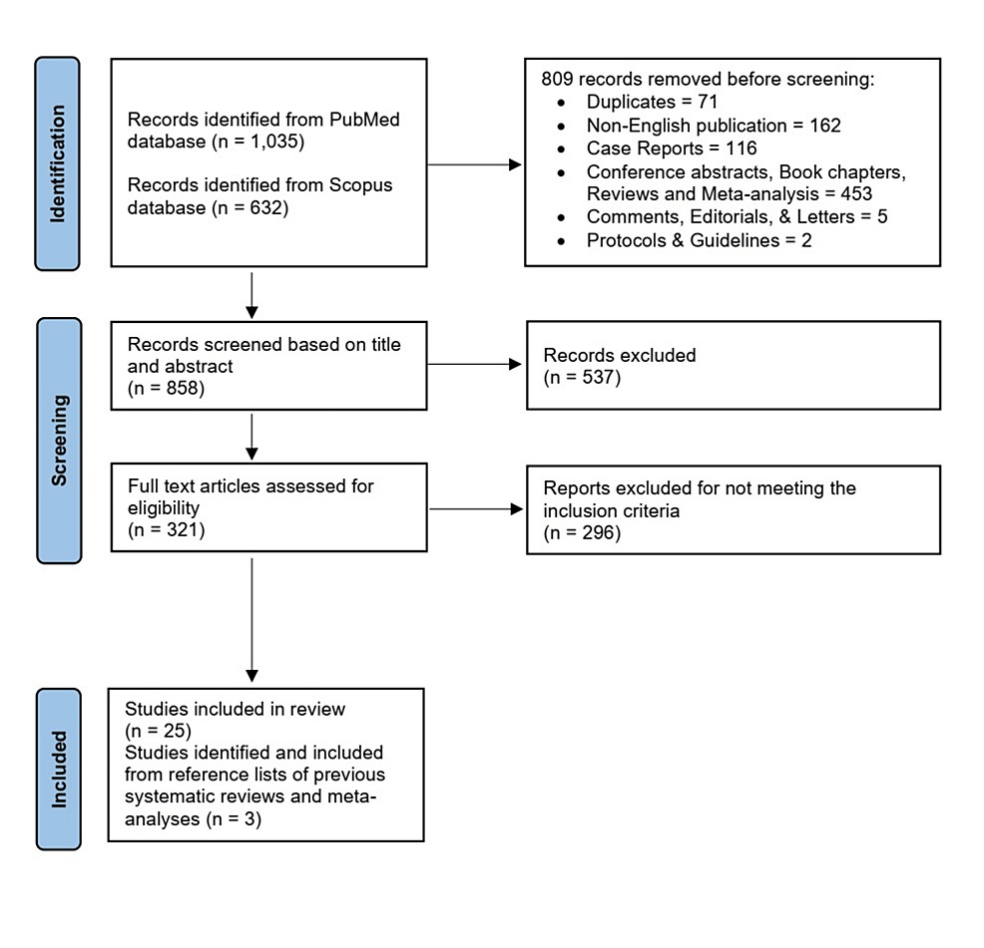
PRISMA flow diagram showing results of the study selection process. PRISMA: Preferred Reporting Items for Systematic Reviews and Meta-Analyses

Our literature search strategy yielded 1,667 papers, of which 809 were removed during prescreening (Figure 1). The title/abstract of the remaining 858 records was screened with full-text screening for 312 records. Finally, 25 articles met the inclusion criteria. In addition, another three articles were identified through the reference lists of previous systematic reviews and meta-analyses. The detailed flow of the study selection process is described in Figure 1.

Study Design, Surgical Approach, and Surgery Location of the Included Studies

Eighteen out of the 28 included studies were RCTs (64.28%) which collectively included 1,183 patients from Australia [23], Brazil [24,25], China [26], Egypt [27], India [28], Iran [29,30], Italy [31,32], Spain [33,34], Turkey [35], and USA [36-40]. The average age of the participants in most RCTs was 50 years or older. In ten RCTs, patients underwent a laparotomy procedure, out of which six specifically involved the large bowel, one involved the small bowel, and three studies did not separately report data from patients who underwent small or large bowel procedures (Table 1).

**Table 1:** Randomized controlled trials reporting tolerance to and/or postoperative outcomes with early oral feeding (EOF) following elective open bowel surgery. TOF = traditional oral feeding; NGT = nasogastric tube; POD = postoperative day. $Gender distribution not reported.

Author (Year)	Country	Participants	Surgery	Feeding Procedure	Tolerance to EOF	Postoperative Complications
Surgery involving large bowel						
Stewart et al., 1998 [23]	Australia	EOF: 40 patients (47.5% females; mean age 58 years (range 25-89 years)). TOF: 40 patients (45% females; mean age 59 years (range 17-88 years)).	Colorectal surgery Specific indication for surgery: No (mixed diagnosis)	EOF: Free fluids after 4h of surgery, progressing to a solid diet from POD1 as tolerated. TOF: Nil by mouth until flatus or defecation, after which fluids were commenced progressing to a solid diet over 24-48h.	80% of patients in the EOF group tolerated solid oral intake within 48 h.	Median time to solid diet (2 vs. 6; p<0.001), full diet (5 vs. 8; p<0.001), flatus (3 vs. 4; p=0.01), first bowel action (4 vs. 5; p=0.03), and discharge (9 vs. 11; p=0.01) were significantly earlier in the EOS group. However, there was no significant difference in the rate of vomiting, NGT reinsertion, prolonged distention, nausea, antiemetic use, or complications.
El Nakeeb et al., 2009 [27]	Egypt	EOF: 60 patients (35% females; mean age 52.3±12.5 years). TOF: 60 patients (30% females; mean age 56.3±11.6 years).	Colonic anastomosis Specific indication for surgery: No (mixed diagnosis)	EOF: Clear liquid diet on POD1 followed by a regular diet as tolerated. TOF: Clear liquid diet after the passage of flatus followed by a regular diet as tolerated.	75% of the patients in the EOF group tolerated early feeding.	Days to first passage of flatus (3.3±0.9 vs. 4.2±1.2; p=0.04), stool (4.1±1.2 vs. 4.9±1.2; p=0.005), and hospital discharge (6.2±0.2 vs. 6.9±0.5; p=0.05) were earlier in the EOF group. Vomiting was more common in the EOF group (25% vs. 16.66%; p=0.05). The incidence of other complications was similar between the two groups except for pulmonary complications, which occurred more frequently in the TOF group (3.33% vs. 11.67%; p=0.05).
Feo et al., 2004 [32]	Italy	EOF^$^: 13 patients (mean age 67.6±10.4 years). TOF^$^: 50 patients (mean age 67.6±10.2 years).	Colorectal resection Specific indication for surgery: Colorectal cancer	EOF: A clear liquid diet on POD1 followed by a soft and solid diet as tolerated. TOF: Clear liquid diet after the passage of flatus followed by a soft then solid diet as tolerated.	Not reported.	Significantly more patients in the EOF groups experienced vomiting (32% vs. 14%) and required NGT insertion (20% vs. 6%) p<0.05 for both. However, the incidence of other complications and the length of hospitalization were similar between the two groups.
Ortiz et al., 1996 [33]	Spain	EOF: 95 patients (40% females; mean age 65.54 years (range 22-90 years)). TOF: 95 patients (42.1% females; mean age 65.7 years (range 39-88 years)).	Colorectal surgery Indication for surgery: No (mixed diagnosis)	EOF: Free intake of clear liquids on the first evening after the operation, followed by regular diet on POD1. TOF: A diet of clear liquids after postoperative ileus resolution advancing to regular diet after 24 hours, as tolerated.	79.6% in the EOF group tolerated early oral intake in the first 4 days (p<0.05 for days 1-4), but no differences between the two groups after POD 4.	The incidence of vomiting and NGT insertion (21.5%) was higher in patients in the EOF group (p<0.05 for days 1-4). However, the time to the first bowel movement (4.3 vs. 4.7 days) and rate of complications (17.3% vs. 19.3%) were similar between EOF and TOF groups.
Dag et al., 2011 [35]	Turkey	EOF: 99 patients (47% females; mean age 62 years (range 35-85 years)). TOF: 100 patients (39% females; mean age 61 years (range 17-89 years)).	Colorectal surgery Specific indication for surgery: Colorectal cancer	EOF: Clear liquid diet ~12 hours after surgery followed by a regular diet as tolerated. TOF: Clear liquid diet after first flatus or stools followed by a regular diet as tolerated.	85.9% of patients in the EOF group tolerated early feeding.	Bowel movements (1.7±0.89 vs. 3.27±1.3 days), defecation (3.4±0.77 vs. 4.38±1.18 days), time of tolerance of solid diet (2.48±0.85 vs. 4.77±1.81 days) and hospital discharge (5.55±2.35 vs. 9.0±6.5 days) (p<0.001 for all) were earlier in the EOF group. However, the rates of NGT reinsertion, overall complication, or anastomotic leakage were similar between the two groups.
Lucha et al., 2005 [39]	USA	51 patients were randomized to EOF and TOF groups. Groupwise age and gender distribution not reported. The average age of the overall study population was 51 years (range 22-74 years); 45% were females.	Colorectal resection Specific indication for surgery: No (mixed diagnosis)	EOF: Regular diet 8 hours after surgery. TOF: 24 hours on clear liquids after the passage of flatus, gradually transitioning to regular diet.	The time to tolerance of a diet was 5.7 and 4.7 days in the TOF and EOF groups, respectively (p=NS).	Postoperative complications and those requiring NGT (19% vs.12%) were similar in the EOF vs. TOF groups, although patients in the EOF group required more postoperative antiemetic therapy (1.7 doses vs. 0.8 doses per patient; p<0.05). The mean length of hospitalization (6.3 vs. 6.6 days) was similar between EOF and TOF groups.
Surgery involving small bowel						
Nematihonar et al., 2019 [29]	Iran	EOF: 54 patients (51% females; mean age 64.10±13.9 years). TOF: 54 patients (55% females; mean age 50.58±18.20 years).	Small intestine anastomosis Specific indication for surgery: No (mixed diagnosis)	EOF: Clear liquid diet on POD1 followed by a regular diet as tolerated. TOF: Clear liquid diet after resolution of postoperative ileus followed by a solid diet as tolerated.	Day of beginning oral feeding in the EOF and TOF groups were 1.0±0.0 and 4.3±0.87, respectively (p˂0.001).	Although first passage of flatus occurred within 2 days in both groups (p=ns), the days to first stool (3.2±0.59 vs. 3.6±0.66; p=0.006), and hospital discharge (3.8±1.06 vs. 6.3±1.0, p= 0.001) occurred earlier in the EOF group.
Surgery involving small or large bowels						
Minig et al., 2009 [31]	Italy	EOF: 18 patients (all females; median age 54 years (range 42-62 years)). TOF: 22 patients (all females; median age 58 years (range 47-66 years)).	Intestinal resection (unspecified) Specific indication for surgery: Gynecologic malignancies	EOF: Clear liquid diet 24 hours after the operation, followed by a regular diet as tolerated. TOF: Clear liquid diet for 24 hours after resolution of postoperative ileus followed by a semi-solid diet as tolerated. Patients transitioned to a regular diet if semi-solid diet was tolerated for 1 day.	78% of patients in the EOF group tolerated solid oral intake on the first POD vs. 0% in the TOF group.	The incidence of nausea and vomiting during the postoperative period was similar between groups (55% vs. 56%). Time to flatus elimination was shorter in the EOF group (2 vs. 3 days; p=0.010), although time to passage of stool (5 vs. 5.5 days), postoperative pain or analgesic requirements, and incidence of overall, infective, or intestinal complications was similar in the two groups. There was a significant reduction in the length of the hospital stay by 1.7 days in the EOF group (p=0.05) after adjusting for the postoperative complication.
Pragatheeswarane et al., 2014 [28]	India	EOF: 60 patients (45% females; mean age 46.5 ± 17.2 years). TOF: 60 patients (47% females; mean age 46.9 ± 16.5 years).	Bowel surgery (unspecified) Specific indication for surgery: No (mixed diagnosis)	EOF: Clear liquid diet on POD1 followed by full fluid diet within 48 h and solid diet over the next 24 h as tolerated. TOF: Oral intake after the resolution of ileus starting on a liquid diet and progressing into solid intake as tolerated.	Time to solid diet was significantly earlier in the EOF group (3.9 ± 2.2 vs. 6.9 ± 3.3; p<0.0001)	The number of days to first flatus (2.6 ± 0.9 vs. 4.5 ± 1.5; p<0.0001), first defecation (3.8 ± 1.3 vs. 6.1 ± 2.1; p<0.0001), and length of postoperative stay (11.1 ± 5.5vs. 14.4 ± 8.5; p=0.011) were significantly earlier in the EOF group. Anastomotic leak, wound infection, fever, vomiting, abdominal distention, and other complications were similar between EOF and TOF groups.
Reissman et al., 1995 [36]	USA	EOF: 80 patients (57.5% females; mean age 51 years (range 16-82 years)). TOF: 81 patients (46.9% females; mean age 56 years (range 20-90 years)).	Colon or small bowel resection Specific indication for surgery: No (mixed diagnosis)	EOF: Clear liquid diet on POD1 followed by a regular diet within the next 24 to 48 hours, as tolerated. TOF: Oral feeding only after postoperative ileus resolution.	79% of the patients in the EOF group tolerated early feeding. Patients in the EOF group tolerated regular diet significantly earlier (2.6±0.1 days) than in the TOF group (5.0±0.1 days; p< 0.001).	No significant differences between the EOF and TOF groups in the rate of vomiting (21% vs. 14%), NGT reinsertion (11% vs. 10%), length of ileus (3.8±0.1 vs. 4.1±0.1 days), length of hospitalization (6.2±0.2 vs. 6.8±0.2 days), or overall complications (7.5% vs. 6.1%) (p = NS for all).

Half of the remaining eight RCTs reported combined data from patients who had undergone either laparotomy or laparoscopy procedures (Table 2), while the other half did not specify the surgical approach (Table 3). In both RCT subgroups, three specifically involved the large bowel, and one involved both small and large bowel procedures (Tables 2-3). We did not identify any RCT that specifically reported the outcomes of EOF after laparoscopic bowel surgery during our study period.

**Table 2:** Randomized controlled trials reporting tolerance to and/or postoperative outcomes with early oral feeding (EOF) following elective open or laparoscopic bowel surgery. TOF = traditional oral feeding; NGT = nasogastric tube; POD = postoperative day

Author (Year)	Country	Participants	Surgery	Feeding Procedure	Tolerance to EOF	Postoperative Complications
Surgery involving large bowel						
Lobato Dias Consoli et al., 2010 [24]	Brazil	EOF: 15 patients (73.3% females; mean age 54.5 years (range 35-75 years)). TOF: 14 patients (64.3% females; mean age 47.4 years (range 21-79 years)).	Colorectal resection Approach: Laparotomy or Laparoscopy Specific indication for surgery: 24 out of 29 patients had colorectal cancer	EOF: 500mL of restricted fluid as the first diet intake on POD1 and regular diet immediately after that if no nausea and vomiting. TOF: Nil by mouth until flatus or defecation.	About 40% of patients tolerated solid diet on POD1 and 80% by POD 3 (0% and 30% in TOF groups, respectively).	Flatus expulsion (1 vs. 2 days) and discharge from hospital (median 3 vs. 5 days) were significantly shorter in the EOF group (p<0.05 for both). However, complication rates and acceptance of diet were similar in both groups. Diarrhea occurred more frequently in the TOF group (OR=1.86; IC 95%:1.08-3.20).
da Fonseca et al., 2011 [25]	Brazil	EOF: 24 patients (66.66% females; mean age 57.4±16.3 years). TOF: 26 patients (61.5% females; mean age 51.7±13.3 years).	Colonic anastomosis Approach: Laparotomy or Laparoscopy Specific indication for surgery: Cancer (unspecified)	EOF: Oral liquid diet on POD1 morning followed by regular diet within 24h as tolerated. TOF: Nothing by mouth until the first flatus and followed by an oral liquid diet and a regular diet within the next 24h.	Tolerance to first oral diet was similar in the two groups (83.3% vs. 80.8%) despite the TOF group initiating oral diet 1.9±0.8 days later. Tolerance to solid diet was numerically higher in the EOF group (95% vs. 71.4%; p=0.093) but was insignificant.	The EOF group's hospital stay was significantly shorter (4.0±3.7 vs. 7.6±8.1 days; p = 0.000). However, the incidence of postoperative complications and mortality was similar between the two groups.
Ortiz et al., 1996 [34]	Spain	Laparoscopy-assisted: 15 patients (53.3% females; mean age 52 years (range 15-77 years)) Laparotomy: 20 patients (46.6% females; mean age 56 years (range 41-74 years))	Colorectal surgery Approach: Laparotomy or Laparoscopy Specific indication for surgery: No (mixed diagnosis)	Patients in both groups initiated on ad libitum clear liquids on the evening of the surgery, and regular diet was provided upon request.	Tolerance to EOF was similar between the two groups between POD 1 and POD 10 (~80% on POD 1).	The incidence of vomiting (~30% in both groups on POD 1 and 0% on POD 7) or NGT insertion (~15-18% on POD 1, 0% on POD 6 with laparotomy, and 0% on POD 10 with laparoscopy; p=NS) was similar between groups. The time to the first bowel movement was 5.4 days with a 26.6% complication rate in the laparoscopy group and 5.5 days with a 13.3% complication rate in the laparotomy group (p=NS for both).
Surgery involving small or large bowels						
Binderow et al., 1994 [40]	USA	EOF: 32 patients (56.2% females; mean age 52 years (range 15-81 years)) TOF: 32 patients (43.7% females; mean age 52 years (range 15-87 years)).	Colon or small bowel resection Approach: Laparotomy and/or laparoscopic-assisted procedure. Specific indication for surgery: No (mixed diagnosis)	EOF: Regular diet on POD1 morning. TOF: 8 oz of ice chips per day immediately after surgery. A diet of clear liquids after postoperative ileus resolution advancing to regular diet after 24 hours, as tolerated.	Not reported.	The proportion of patients experiencing vomiting (44% vs. 25%; p<0.05) was higher in the EOF group than in the TOF group. The proportion of patients requiring NGT reinsertion for distention with persistent vomiting, duration of postoperative ileus, and length of hospitalization was similar between the two groups.

**Table 3:** Randomized controlled trials reporting tolerance to and/or postoperative outcomes with early oral feeding (EOF) following bowel surgery with unspecified surgical approach. TOF = traditional oral feeding; NGT = nasogastric tube; POD = postoperative day. $Gender distribution not reported

Author (Year)	Country	Participants	Surgery	Feeding Procedure	Tolerance to EOF	Postoperative Complications
Surgery involving large bowel						
Zhou et al., 2006 [26]	China	EOF: 161 patients (42.8% females; mean age 55.3±16.7 years). TOF: 155 patients (46.5% females; mean age 57.1±19.8 years).	Colorectostomy Approach: Not reported. Specific indication for surgery: Colon or rectal cancer	EOF: The NGT was removed after 12 to 24 h followed by oral feeding. TOF: The NGT was removed after the passage of flatus, followed by oral feeding.	Not reported.	Days to the first passage of flatus (3.0±0.9 vs. 3.6±1.2; p<0.001), the first passage of stool (4.1±1.1 vs. 4.8±1.4; p<0.001), and hospital discharge (8.4±3.4 vs. 9.6±5.0; p<0.05) were earlier in the EOF group. The postoperative complications such as anastomotic leakage, acute dilation of stomach, and wound complications were similar in the groups, but fever (3.73% vs. 9.68%; p<0.05), pulmonary infection (0.62% vs. 4.52%; p<0.05), and pharyngolaryngitis (3.11% vs. 23.23%; p<0.001) occurred more frequently in the TOF group.
Nematihonar et al., 2018 [29]	Iran	EOF: 30 patients (43.3% females; mean age 45.8 ± 17.1 years). TOF: 30 patients (40% females; mean age 46.8 ± 13.6 years).	Colorectal anastomosis Approach: Not reported. Specific indication for surgery: No (mixed diagnosis)	EOF: Clear liquid diet within 24 hours of surgery followed by a regular diet as tolerated. TOF: Clear liquid diet after resolution of postoperative ileus followed by a solid diet as tolerated.	93% tolerated early feeding in the EOF group.	The days to ileus resolution (2.5±0.7 vs. 3.4±0.9; p<0.0001), first passage of flatus (2.66±0.71 vs. 3.9±0.71; p<0.0001), defecation (3.9±0.92 vs. 5.4±0.77; p<0.0001), and hospital discharge (4±0.64 vs. 6.1±0.84; p<0.0001) were shorter in the EOF group. However, pain scores were significantly higher in the EOF group (8.6 ± 1.2 vs. 7.1 ± 1.6; p<0.0001). Other complications such as nausea, vomiting, NGT insertion, prolonged distention, infections, fever, anastomosis leakage, and reoperation were similar between the two groups.
Hartsell et al., 1997 [37]	USA	EOF^$^: 29 patients (mean age 66 years (range 22-82 years)). TOF^$^: 29 patients (mean age 68 years (range 40-83 years)).	Colorectal surgery Approach: Not reported. Specific indication for surgery: No (mixed diagnosis)	EOF: Full liquid diet on POD1 advancing to regular diet on POD2 If the patient consumed ≥1000 in 24 hours. TOF: Similar diet plan as the EOF groups but only on return to normal bowel function with the passage of flatus or stool.	All patients in the EOF group tolerated the liquid diet on POD1.	The difference in the occurrence of nausea (55% vs. 50%) or vomiting (48% vs. 33%) and those requiring NGT (27% vs. 16%) in the EOF vs. TOF groups were statistically not significant. The rate of complications and the mean length of hospitalization (7.2±3.3 vs. 8.1±2.3 days) were also similar between EOF and TOF groups.
Surgery involving small or large bowels						
Behrns et al., 2000 [38]	USA	EOF: 27 patients (44% females, mean age 45±3 years). TOF: 17 patients (59% females, mean age 47±4 years).	Small bowel or colonic resection Approach: Not reported. Specific indication for surgery: No (mixed diagnosis)	EOF: 30ml of clear liquids per hour on POD2, unlimited clear liquids on POD3, and discharge on POD 4 with dietary instructions to start a solid diet immediately upon returning to the home environment. TOF: Clear liquids after the passage of stool or flatus which was gradually increased until patients tolerated a total volume of ~750 ml without nausea, vomiting, or abdominal distention. Solid diet was subsequently initiated and discharged from the hospital after three solid meals.	Not reported.	The difference in postoperative complication rates (19% vs. 29%) in the EOF vs. TOF groups was statistically insignificant. Postdischarge symptoms (nausea, vomiting, constipation, diarrhea, low urine output, light-headedness, and dietary intolerance) occurred in 82% of patients in the TOF group and 56% in the EOF group (p=0.1) on day 1, and 6% and 30% on day 3 (p=0.12). The mean length of hospitalization (4.4±0.2 vs. 6.1±1.1 days; p=0.09) was also similar between EOF and TOF groups.

Additionally, we identified six PCS [41-46] reporting tolerance to and/or postoperative outcomes with EOF, all of which involved the large bowel surgery (Surgery approach: Laparotomy = 3, Laparoscopy = 1, Laparotomy or laparoscopy = 1, and Unspecified = 1; Table 4). Collectively these studies included data from 750 patients from four countries.

**Table 4:** Prospective case series reporting tolerance to and/or postoperative outcomes with early oral feeding (EOF) following elective bowel surgery. TOF = traditional oral feeding; NGT = nasogastric tube; POD = postoperative day

Author, Year	Country	Participants	Type of Surgery	Feeding Procedure	Tolerance to EOF	Post-operative Complications/Predictors
Di Fronzo et al., 1999 [41]	USA	200 patients (48% females, mean age 63.1 years (range 25-88 years)).	Colectomy Approach: Laparotomy Specific indication for surgery: No (mixed diagnosis)	Clear liquid was orally administered on the evening of POD2, regular diet on POD3, and discharged home as tolerated.	86.5% of the patients tolerated EOF.	4.5% of patients experience postoperative complication including wound infection, pancreatitis, perineal wound dehiscence, and hemorrhage. Gender (male) and type of operation (total abdominal colectomy or total proctocolectomy) were associated with failed EOF while age, comorbid medical illness, operative time, or additional surgical procedures were not.
DiFronzo et al., 2003 [42]	USA	87 elderly patients (52% females; mean age 77 years (range 70-90 years)).	Colon resection Approach: Laparotomy Specific indication for surgery: No (mixed diagnosis)	All patients initiated clear liquids on POD2, regular diet on POD3, and discharge to home as tolerated.	89.6% of patients tolerated early feeding.	The mean hospital stay was 3.9 days (range: 3-12 days). 14.9% of patients experienced postoperative complications. However, there were no instances of pneumonia, abscess, anastomotic leak, and perioperative deaths. 83% of patients were discharged to home by POD 4. 96.5% of patients who were previously independent for daily functions did not require assisted care after surgery.
Detry et al., 1999 [43]	Belgium	33 patients (54.5% females, mean age 52 years (range 20-77 years)).	Colorectal surgery Approach: Laparotomy Specific indication for surgery: No (mixed diagnosis)	NGT was removed 12-18 hours of surgery and oral feeding was resumed 4 hours later with liquids and a slight solid diet was started at the first next meal.	Tolerance was excellent in 66% of the patients, good (slight complaints) in 16%, while the diet had to be stopped for few hours in 9% of the patients and another 9% required NGT insertion.	There was no postoperative mortality or significant morbidity. Intestinal transit was observed after a median period of 2 days and postoperative stay ranged from 5 to 12 days (median: 7 days). The type of surgery did not predict tolerance to EOF although postoperative stay was significantly shorter after colostomy closure than after anterior resection and abdominal perineal resection (p<0.005), and for patients who tolerated EOF (7.3 days vs. 10.5 days; t = -6; p<0.001).
Leung et al., 2022 [44]	Canada	221 patients (38.9% females, mean age 63.4±15 years).	Colectomy without stoma Approach: Laparoscopy Specific indication for surgery: No (mixed diagnosis)	Patients were given sips of water after recovery from general anesthesia. A clear liquid diet (300mL) was provided upon transfer to the surgical ward, and then transitioned to regular diet regardless of bowel function on POD1.	69% of the patients tolerated clear liquid diet on POD0.	The mean time to tolerating solid intake (29.9±28.3 vs. 53.5±8 hrs.; p=0.003), flatus elimination (26.4±12 vs. 32.1±24.1 hrs.; p=0.019) and GI-3 score (tolerating solid diet and passage of gas or stools; 34.5±28.5 vs. 50±63.4 hrs.; p=
Fujii et al., 2014 [45]	Japan	EOF (POD1): 62 patients (41.69% females; mean age 67.4±11.7 years). EOF (POD2): 58 patients (48.27% females; mean age 66.9±10.7 years).	Colorectal surgery Approach: Laparotomy or Laparoscopy Specific indication for surgery: Colon or rectal cancer	EOF (POD 1): Full liquid diet on POD1 advancing to regular diet within 24-hour period. EOF (POD 2): Full liquid diet on POD2 advancing to regular diet within 24-hour period.	Patients in the POD1 group tolerated liquid (1.2±0.7 vs. 2.3±0.6 days) and solid (2. ±0.8 vs. 3.5±0.8 days) diets significant earlier than patients in POD2 group (p<0.001 for both).	The mean time to flatus elimination (2.3±0.7 vs. 3.1±1.0 days) and defecation (3.2±1.2 vs. 4.2±1.4) was shorter among patients in POD1 than the POD2 group (p<0.001 for both). Postoperative morbidity, mortality, or duration of hospital stay (9.6±4.6 vs. 9.6±6.3 days) were similar between the two groups.
Petrelli et al., 2001 [46]	USA	89 patients (50% females, mean age 65 years (range 28-87 years)).	Colectomy Approach: Not reported Specific indication for surgery: No (mixed diagnosis)	A full liquid diet was initiated on either POD1 (21 patients), POD2 (64 patients), or POD3 (3 patients).	73% of the patients tolerated EOF.	The amount of estimated blood loss was the only variable that was significantly associated with successful early oral postoperative feeding, while a null association was observed with age, sex, ASA score, epidural analgesia use, operating time, side of surgery, previous abdominal surgery, additional procedures performed, colloid volume, oral intake with initial feeding, day of initial feeding, and crystalloid fluid requirements.

Finally, four cohort studies [47-50] met our inclusion criteria and were included in this review; all four involved large bowel surgery (Surgery approach: Laparotomy = 1, Laparotomy or laparoscopy = 3; Table 5). Collectively, the cohort studies included data from 714 patients from three countries.

**Table 5:** Cohort studies reporting tolerance to and/or postoperative outcomes with early oral feeding (EOF) following elective bowel surgery. TOF = traditional oral feeding; NGT = nasogastric tube; POD = postoperative day. $Gender distribution not reported

Author, Year	Country		Participants	Type of Surgery	Feeding Procedure	Tolerance to EOF	Post-operative Complications/Predictors
Prospective cohort studies							
Choi et al., 1996 [47]	USA		EOF: 41 patients (68.3% females; mean age 64.5 years (range 16-88 years)). TOF (historical controls): 41 patients (68.3% females; mean age 67.1 years (range 39-84 years)).	Colon resection Approach: Laparotomy Specific indication for surgery: No (mixed diagnosis)	EOF: Clear liquid diet on POD2, regular diet on POD3, and discharge to home as tolerated. TOF: Enteral diet after return of bowel sound and the passage of flatus and/or bowel movements.	90% of patients tolerated early feeding in the EOF group.	Fewer patients in the EOF group experienced vomiting (9.7% vs. 14.6%). As a result, none of the patients in EOF group required NSG tube but was required in 9.8% of patients in the TOF group. The mean hospital stay was 4.2 days (range: 3-8 days) in in the EOF group versus 6.7 days (range: 5-34 days) in the TOF group. 67% of patients in the EOF were discharged home by POD4 whereas all patients in the TOF group were discharged on POD5 or later. There was no readmission for within 2 weeks for recurrent nausea or vomiting in both groups.
Kawamura et al., 2000 [48]	Japan		EOF^$^: 22 patients (mean age 62.2±2.1 years). TOF^$^: 155 patients (mean age 62.7±2.5 years).	Colorectal surgery Approach: Laparotomy or Laparoscopy (Most patients in EOF group allocated to laparoscopic procedure) Specific indication for surgery: Colon or rectal cancer	EOF: The NGT was removed on POD1 morning immediately followed by fluid intake. A liquid meal was initiated from POD3 morning transitioning to semi-solid diet by POD5 and regular diet on POD6. TOF: The NGT was removed on POD1 morning and after passage of flatus or stool, similar diet plan as the EOF group.	Not reported.	Postoperative stay was shorter in the EOF group than TOF group (11.5±1.2 vs. 24.0±2.1 days; p<0.0001). The incidence of postoperative complications and mortality was similar between the two groups.
Gianotti et al., 2011 [49]	Italy		EOF: 100 patients (43% females; mean age 61.2 ± 8.1 years). TOF (historical controls): 100 patients (39% females; mean age 59.8 ± 10.6 years).	Colorectal resection Approach: Laparotomy or Laparoscopy Specific indication for surgery: No (mixed diagnosis)	EOF: Oral nutritional supplement (ONS) on POD1. Normal food plus ONS to reach 1,000-1,200 kcal/day on POD 2 with progressive increase until 1,800-2,000 kcal/day. TOF: Oral feeding after full bowel function recovery	Early oral feeding was tolerated by 67% of the patients (with no side effects) by POD2 and 89% by POD5.	The occurrence of postoperative nausea, vomiting, diarrhea, abdominal distension, NGT reinsertion or other complications was similar between the two groups. However, the median length of hospitalization was shorter in the EOF group (9 days vs. 12 days; p = 0.01).
Retrospective cohort studies							
Toledano et al., 2019 [50]		USA	EOF: 204 patients (46.6% females; mean age 55.6±16.9 years (range 19-93 years)). TOF: 51 patients (54.9% females; mean age 60.0±16.8 years (range 19-92 years)).	Colorectal surgery with ileostomy or colostomy Approach: Laparotomy or Laparoscopy Specific indication for surgery: No (mixed diagnosis)	EOF: Liquid diet or a low-fiber diet (≤10 grams fiber per day) on POD0 or POD1. TOF: Liquid diet or a low-fiber diet on POD2 or later.	Not reported.	Time to first flatus (2.4±1.4 vs. 3.3±1.4 days; p<0.001) and first ostomy (2.3±1.3 vs. 3.5±1.6 days; p<0.001) were significantly shorter in the EOF group. These associations remained significant after adjusting for ASA physical status classification, resection type, type of ostomy created, and surgical approach. The median length of stay was significantly shorter in the EOF group (6 vs. 9 days; p<0.001).

Post-surgery Diet Resumption Plan of the Included Studies

EOF in 15 RCTs was initiated with a liquid diet on POD 0 or 1, transitioning to a semi-solid or regular diet irrespective of the passage of flatus or stool. However, three studies initiated patients on a regular diet after 8-24 hours of bowel rest [26,39,40]. The control group in all RCTs followed the TOF regime.

There was more heterogeneity with the initiation of oral feeding among the PCS and cohort studies. For example, one PCS initiated EOF on POD 0 [44], another at POD 1 [43], two on POD 2 [41,42], and another two included patient groups who started EOF on POD 1, 2, or 3 [45,46]. Similarly, in one cohort study, EOF was started on POD 0 or 1 [50], two initiated feeding on POD 1 [48,49], and one on POD 2 [47]. In all PCS and cohort studies included in this review, EOF was initiated with a liquid diet and the subsequent introduction of a regular diet.

Tolerance to EOF

Thirteen out of the 18 RCTs reported tolerance to EOF on different PODs. Among patients who underwent open large bowel surgery, tolerance to a regular diet was reported in 85.9% of patients on POD 1 [35] and 75% to 95% between POD 2 and 3 [23,27,33] (Table 1). Lucha et al. (2005) [39] reported that patients who underwent open large bowel surgery tolerated a regular diet on an average of 4.7 days on EOF modality compared to 5.5 days of TOF patients, albeit non-significantly. Similarly, high tolerance to EOF was reported among patients who underwent an open small bowel procedure [30] or an unspecified open bowel procedure [28,31,36], with patients in the EOF groups tolerating regular diet between POD 2 and 3 compared to POD 5 to 7 in TOF groups [28,36] (Table 1).

Consistent with these observations, RCTs that reported combined outcomes from patients who underwent either laparotomy or laparoscopy bowel procedures [24,25,34] (Table 2) or did not specify the surgery approach [29,37] (Table 3) also suggest that most patients tolerate EOF with liquid and subsequent solid diet. It is noteworthy that Ortiz et al. (1996) [34] showed a comparable tolerance to EOF (~80% on POD 1) among patients who underwent laparotomy or laparoscopy colorectal surgery (Table 2).

Postoperative Side Effects and Complications

In most RCTs, there were no differences in the occurrence of vomiting [23,28,29,31,36-38], nausea [23,28,29,31,37,38], or patients requiring nasogastric tube (NGT) [23,28,29,35-37,39,40] between EOF and TOF groups, while others suggested an increased occurrence of vomiting [27,32,33,40], nausea [33], and NGT requirement [32] with EOF. None of the studies showed any advantage of EOF over TOF in terms of vomiting, nausea, and NGT requirement. Similarly, eight RCTs showed an earlier return of bowel function with EOF [23,26-31,35], while four reported no differences between EOF and TOF groups [24,33,36,40]. One study showed lower odds of postoperative occurrence of diarrhea with EOF than TOF [24], while another reported no differences with either feeding regime [38].

In terms of length of hospital stay, patients with EOF were discharged significantly earlier than patients with TOF in 10 of the included RCTs [23-31,35], while six RCTs reported a similar length of hospitalization with early or traditional feeding [32,36-40]. Furthermore, 15 of the 17 included RCTs consistently showed no differences in the overall rate of postoperative complications with EOF and TOF [23-29,31-33,35-39] (two RCTs did not report complication rates). It is noteworthy that EOF patients had lower incidences of pulmonary complications in two studies than TOF patients [26,27], while EOF patients had higher pain scores in one study [29] but were similar to TOF patients in another [31]. Similarly, the findings of the cohort studies were consistent with RCTs in showing that although the occurrence of nausea, vomiting, or other complications was comparable between EOF and TOF, patients with EOF had a shorter length of hospitalization [47-50].

Although RCTs investigating the tolerance to oral feeding on POD 0 are scarce, one PCS suggests that patients who tolerate oral feeding on POD 0 might have better postoperative outcomes in terms of return of bowel function, earlier tolerance to a solid diet, in-hospital complications, or the length of hospitalization [44]. However, all patients in this PCS [44] were operated on with laparoscopy, and there is no direct evidence that patients who undergo an open procedure and tolerate oral feeding on POD 0 also have better postoperative outcomes.

The amount of blood loss [46], male gender [41], and type of operation (total abdominal colectomy or total proctocolectomy) [41] have been identified as factors determining early tolerance to oral feeding. Moreover, the type of surgery may also predict a shorter hospital stay among EOF patients [43]. However, age, comorbidities, operative time, and additional surgical procedures were not associated with early tolerance to oral feeding or better postoperative outcomes [41,46].

Discussion

The currently available evidence suggests that although many patients may be able to tolerate oral feeding by POD 2 or 3, about 5-25% of patients do not tolerate oral feeding until POD 4, which is also typically when the first flatus and the resolution of ileus occurs, and patients in the TOF begin to tolerate oral feeding [23,25,29,30,35,36,39]. Moreover, the benefits of EOF on postoperative outcomes in patients undergoing bowel surgery may be marginal compared to TOF. For instance, EOF, at best, has no advantage over TOF in terms of vomiting, nausea, and NGT requirement and, at worst, may increase vomiting and nausea. Furthermore, all studies consistently show similar postoperative complication rates with EOF and TOF. However, the evidence for early return of bowel function, lower risk of postoperative diarrhea, and lower pain score with EOF are inconsistent, and shorter hospital stays with EOF may be limited to patients who can tolerate oral feeding on POD 0 or 1. Finally, there is some evidence to indicate that the type of surgery and blood loss during surgery may be a determinant of early tolerance to oral feeding, and it remains plausible that tolerating oral feeding early and improved postoperative outcomes, if any, may both be downstream of the type and events during surgery.

Notably, 79% of the included studies reported patient outcomes with EOF following large bowel surgery, and the literature is less extensive with small bowel procedures. Similarly, laparotomy was the surgical approach in 46% of the studies, while the rest combined patients who underwent laparotomy or laparoscopy procedures. Only two studies specifically reported patient outcomes with laparoscopy, one of which compared patient outcomes with EOF following laparotomy or laparoscopy procedures. Therefore, there is insufficient literature to conclude whether EOF has comparable outcomes following laparotomy or laparoscopy bowel surgery.

Nonetheless, the findings of this study are consistent with earlier systematic reviews. For example, McAlee et al. and Zhuang et al. showed that EOF in patients who underwent elective colorectal surgery was associated with earlier resolution of postoperative ileus and hospital discharge without a statistically significant increase in nausea, vomiting, or reinsertion of an NGT even though this conclusion was based on the review of only five [18] and seven [17] studies respectively. A more exhaustive Cochrane review compared early nutrition, orally or through any kind of tube feeding, with traditional nutrition of nil-by-mouth until ileus resolution among patients undergoing any type of lower GI surgery also noted no differences in adverse events and complication rates, and although early feeding was associated with shorter hospitalization, there was considerable heterogeneity in reporting [15]. Nevertheless, a shorter hospital stay in EOF patients could significantly lower hospitalization costs [51].

Our study design allowed the inclusion of more studies than previous systematic reviews in this domain, which enabled a more thorough comparative analysis. However, our literature review was limited by the paucity of available information on several aspects of EOF after bowel surgery. First, only a few studies initiated EOF with solid or regular diets. In 90% of the included studies, feeding was initiated conservatively with a clear liquid diet, even though earlier studies indicate similar outcomes with the early introduction of liquid or solid diets after GI surgery. For instance, in an RCT where one group was initiated on an oral liquid diet and the other on a regular diet soon after the removal of the NGT, there was no significant difference in the occurrence of vomiting, abdominal distention, intestinal obstruction, acute gastric dilatation, or the overall complication rate [52]. More recently, a low-residue diet has been shown to reduce adverse effects and complication rates after colorectal surgery compared to a clear liquid diet [53]. However, initiating early feeding with a liquid diet may also have merits. For example, early intake of warm water was associated with early flatulence among patients who underwent laparoscopic cholecystectomy and were randomized to drink warm water in the fourth postoperative hour or nil by mouth until the eighth postoperative hour when patients in both groups initiated oral fluids and soft food [54].

Second, while the type of surgery is associated with tolerance to EOF, only two studies investigated such an association. Third, we could not ascertain if the responses to EOF varied by indication for the surgery; most studies presented data from cohorts of patients with a mixed diagnosis, and when responses to EOF were reported in patients with a specific condition, it was usually colorectal cancer. If EOF is tolerated or produces a superior outcome compared to TOF in patients with, for instance, Crohn’s disease, diverticulitis, or inflammatory bowel disease is currently unknown. Finally, there was a notable absence of studies comparing EOF and TOF in patients who underwent bowel surgery with laparotomy or complete laparoscopy. In the one study we identified, there was no difference in tolerance to EOF, adverse effects, or complications between patients who had undergone laparoscopic or open bowel surgery [34].

## Conclusions

The current evidence indicates that < 25% of patients may not be able to tolerate EOF after bowel surgery until POD 4. However, patients who tolerate EOF may have a shorter length of hospitalization which may be associated with a lower cost of hospitalization. Therefore it is imperative to investigate factors associated with tolerance to EOF. A handful of available studies have identified blood loss, gender, and type of procedure but not age, comorbidities, operative time, or additional surgical procedures as factors determining early tolerance to oral feeding. Also, most studies report similar adverse effects and postoperative complication rates with EOF and TOF, and healthcare professionals must weigh the benefits of EOF with medical resource availability and/or utilization for implementing EOF. However, it is important to note that the differences in the studies' design, setting, and population may not permit generalization. Therefore, a meta-analysis of this study would provide greater insight into the results of this study. Further studies should be conducted with identical baseline conditions to verify the results of this study.
